# Unraveling Lithium Dynamics in Solid Electrolyte Interphase: From Graph Contrastive Learning to Transport Pathways

**DOI:** 10.1021/acs.jctc.6c00987

**Published:** 2026-09-09

**Authors:** Qiye Guan, Yongqing Cai

**Affiliations:** Institute of Applied Physics and Materials Engineering, 59193 University of Macau, Taipa, Macau 999078, China

## Abstract

Fast lithium transport across the solid-state electrolyte (SSE)/lithium metal anode interface is critical for high-performance all-solid-state batteries. Uncovering the complex lithium dynamics governed by diverse local environments in the solid electrolyte interphase (SEI) is fundamental for performance optimization. A general framework for characterizing the distinct local environments remains lacking, which largely prevents the unlocking of mechanisms governing complex lithium dynamics in the SEI. Here, we develop Graph-based Estimation of ionic Transport in SEI (GET-SEI), a general framework that discovers local atomic environments without predefined labels through graph contrastive learning (GCL). Based on this scheme, lithium transition kinetics can be modeled and quantified via extended dynamic mode decomposition and transition path theory. Applied to various well-known SSE/Li systems, including sulfides (Li_6_PS_5_Cl/Li, Li_10_GeP_2_S_12_/Li) and oxides (Li_7_La_3_Zr_2_O_12_/Li), GET-SEI identifies dominant transport pathways and kinetic bottlenecks in each system, providing quantitative metrics for evaluating lithium transport efficiency. As novel high-performance SSEs continue to emerge, GET-SEI offers a widely applicable, interpretable tool for targeted SEI engineering.

## Graphical abstract

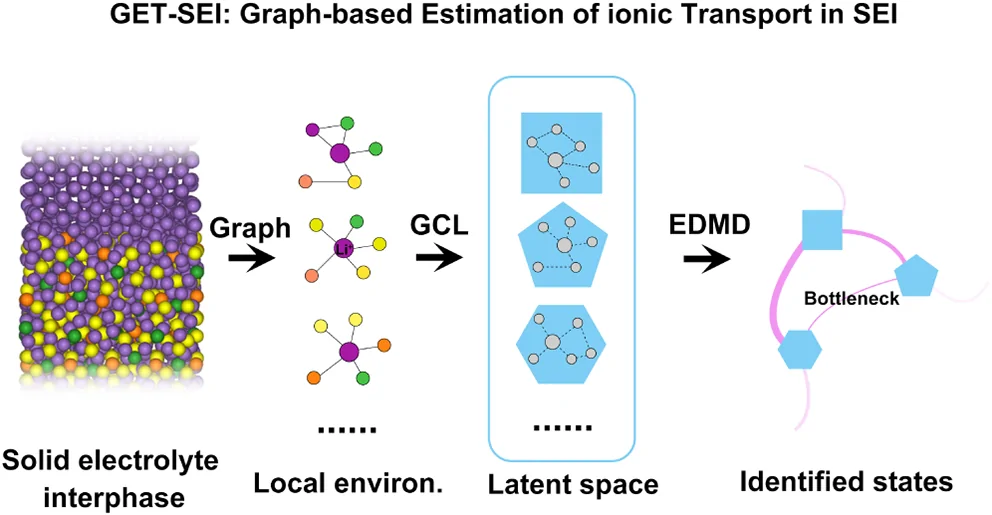

## Introduction

The development of all-solid-state batteries has reached a critical stage, driven by the promise of high energy density and improved safety.^1^ Substantial efforts have focused on discovering new solid-state electrolytes (SSEs) with high ionic conductivity, compatibility with lithium metal, and electrochemical stability.^2–4^ At the same time, understanding the solid electrolyte interphase (SEI) is equally important, because this interphase governs interfacial lithium transport, dendrite suppression, and parasitic side reactions.^5–14^ Unfortunately, the structures within the SEIs are highly complex and disordered due to the varying reactivity and voltage stability windows of different SSEs. Given the abundant and diverse local environments in amorphous SEIs,^15–17^ characterizing lithium dynamics across these environments is essential for understanding and designing interphases that enable rapid and stable interfacial transport.^8,18^

Experimentally, techniques such as X-ray photoelectron spectroscopy (XPS), scanning electron microscopy (SEM), and operando X-ray computed tomography have provided direct evidence of SEI formation and dendrite evolution.^10,13,15^ However, lithium dynamics at the microscopic level within the SEI remain insufficiently resolved. Molecular dynamics and Monte Carlo simulations can capture aspects of the formation and structural evolution of the SEIs,^19–22^ but extracting interpretable lithium transport dynamics from these high-dimensional trajectories remains challenging. Analyses based on representative intermediates offer useful qualitative insights, yet often oversimplify dynamic transport processes.^5,19,23,24^ Therefore, a computationally efficient and generalizable framework that captures complex lithium dynamics is needed for predictive modeling and rational interphase design of the SEIs.

Machine learning has achieved significant progress in terms of physics-informed neural networks for modeling complex dynamics. Although deep kinetic learning methods^25,26^ (e.g., VAMPnets^27,28^ and graph dynamical networks^29^) can capture complex transition patterns, they typically provide latent coordinates with limited physical interpretability.^30–32^ In SEI systems, where lithium transport is controlled by diverse and system-specific local environments, this limitation prevents linking learned dynamics to physically meaningful local environments. If local environments are identified as interpretable states, their transitions can be projected onto reduced Koopman models^33,34^ via methods like extended dynamic mode decomposition (EDMD)^35^ to obtain tractable, approximately linear dynamics for quantitative analysis. Identifying representative local chemical environments and quantifying their similarity is therefore the central challenge. Graph-based deep learning provides a natural representation of atomistic structures and has been successfully applied to universal force-field development^36,37^ and structure-property prediction.^38–41^ For state discovery and classification, graph contrastive learning (GCL) offers an efficient self-supervised framework for learning similarity across multiple graph scales.^42,43^ This makes GCL particularly suitable for abundant Li-centered micro-environment graphs in SEI systems, enabling robust and accurate discovery of physically meaningful local-state relationships.

Here, we establish Graph-based Estimation of ionic Transport in SEI (GET-SEI), a general pipeline that integrates molecular dynamics and graph-based deep learning to model and analyze the complex dynamics of lithium atoms in the SEI. Applied to representative SEI systems, including Li_6_PS_5_Cl/Li (LPSCl/Li), Li_10_GeP_2_S_12_/Li (LGPS/Li) and Li_7_La_3_Zr_2_O_12_/Li (LLZO/Li), the GET-SEI demonstrates both computational efficiency and cross-system generalizability. Unlike black-box latent models, the GET-SEI yields physics-informed learning with mechanistically interpretable, state-based representations that are directly linked to local structural features. Extended dynamic mode decomposition (EDMD) and subsequent transition path theory (TPT)^44,45^ analysis further identify rate-limiting transitions and dominant escape pathways, providing quantitative insight into lithium conduction and a practical framework for the design and evaluation of the SEIs.

## Results and Discussion

### Identify Local Environments in SEIs through Graph Contrastive Learning

Unlike periodically ordered inorganic SSEs or lithium metal phases, amorphous SEIs and their associated lithium transport dynamics are intrinsically difficult to model because of the abundance and diversity of local environments. Each mobile lithium atom can exhibit a distinct local fingerprint that is not fully described by bond topology or static atomic coordinates alone. To address this complexity, we represent each Li-centered neighborhood as a local graph, G=(V,E) , where vertices *V* denote atoms and edges *E* encode local connectivity (Figure 1a). The atomic node entails inclusive environmental features, such as coordination descriptors, average neighbor distances, and local positional statistics, capturing subtle but physically relevant environmental variations.

**Figure 1:**
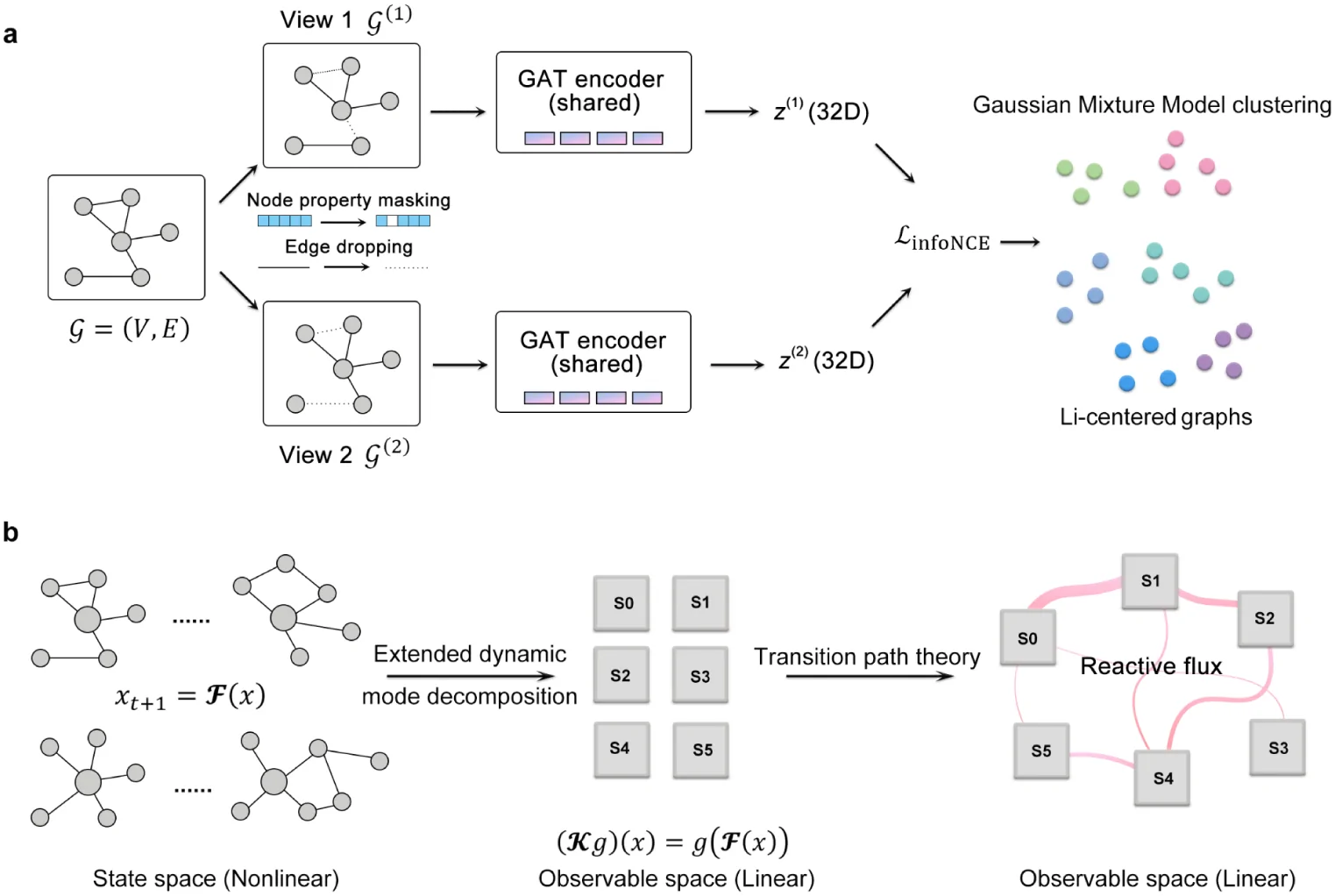
GET-SEI framework for analyzing lithium dynamics in solid electrolyte interphases. **a**, Graph contrastive learning model in encoding and classifying local environments of Li atoms in SEIs. Two augmented graphs $G^{\left(1\right)}$ and $G^{\left(2\right)}$ are sampled from the input graph G by random-edge dropout and random node-feature masking. A graph attention network (GAT) encoder and a projection head are trained to maximize the agreement between representations z^(1)^ and z^(2)^ via Information Noise-Contrastive Estimation (infoNCE) loss. The final Li-centered graphs are classified through a Gaussian mixture model. **b,** Modeling of lithium dynamics through extended dynamic mode decomposition and lithium flux analysis through transition path theory. The S0 to S5 denotes lithium states identified by graph contrastive learning.

We then employ graph contrastive learning (GCL) to compare and classify these local environments. As shown in Figure 1a, input graphs are constructed from neural-network molecular dynamics (NNMD) trajectories (see Methods for details). The core principle of contrastive learning is to maximize agreement between representations of the same sample under suitable augmentations.^46^ Specifically, each graph G is transformed into two correlated views, $G^{\left(1\right)}$ and $G^{\left(2\right)}$ , using random edge dropout and random node-feature masking. These augmented graphs are encoded by a scalar-invariant graph attention network (GAT)^42,47^ with two multi-head attention layers (4 heads). Each node is represented by scalar attributes and message passing aggregates neighboring information in a non-directional manner. The encoder output is projected into a 32-dimensional embedding space.

To quantify graph similarity, we train the GCL model using the information Noise Contrastive Estimation loss $\left(L_{\mathrm{infoNCE}}\right)$ :^42^

1 $$L_{\mathrm{infoNCE}}=-\log\frac{\exp\left(f{(z}^{\left(1\right)}{,z}^{\left(2\right)}\right)/\sigma )}{\overset{N}{\underset{j=1}{\sum }}\exp\left(f\left(z^{\left(1\right)}{,z}^{\left(2\right)}\right)/\sigma \right)}$$

where $z^{\left(1\right)}$ and $z^{\left(2\right)}$ denote the embeddings of the same Li-centered local graph from the two augmented views, $G^{\left(1\right)}$ and $G^{\left(2\right)}$ , respectively. *f* is the cosine similarity function, *N* is batch size, and σ is the parameter controlling the sharpness of the contrastive distribution. This self-supervised objective organizes the embedding space such that similar local environments are mapped nearby, while dissimilar environments are separated. The learned embeddings are then clustered using a Gaussian mixture model (GMM) with an optimally selected number of components, enabling identification of dominant local-environment states in SEI systems.

### Modeling Lithium Dynamics through EDMD and TPT

Having classified local environments in the SEIs via GCL, we next apply the Koopman theory^33^ to represent the generally nonlinear dynamics of SEI systems in a linear framework. Instead of tracking how states evolve, this approach monitors how observables (i.e., scalar functions of states) evolve. For a dynamic system with flow map F , the state update follows:

2 $$x_{t+1}=F\left(x_{t}\right)$$

where *x_t_* is the system state at the *t* moment and *x_t_* lies in the original high-dimensional chemical space M , and full atomic velocities/positions need to be considered. Directly modeling dynamics in M is computationally challenging, so we introduce Koopman operator K , which acts linearly on observable g:M→R . Specifically, K maps the observable *g* to a new function Kg such that evaluating the new function at the current system state *x* (*x*_*t*_) gives the observable’s value at the next state (*x_t_*_+1_):

3 (Kg)(x)=g(F(x))

In this way, a finite-dimensional nonlinear dynamical system is equivalently described by a linear, infinite-dimensional operator K acting on observables.

Using extended dynamic mode decomposition (EDMD),^35^ instead of getting the theoretical infinite-dimensional operator on the full observable space, we derive a finite-dimensional approximation of K matrix from trajectory data (details in Methods). The equilibrium population of each state is obtained from the left eigenvector of K with eigenvalue λ=1 . We then compute the continuous-time rate matrix ***R*** via K=exp(R·τ) :

4 R=ln⁡K/τ

where τ is the lag time. Off-diagonal entries *R_ij_* (*i* ≠ *j*) are transition rates from *i* to *j*. The escape rate of state *i* is −*R_ii_*. To move beyond rate magnitudes and resolve mechanistic pathways, we perform transition path theory (TPT).^44^ The reactive flux $f_{ij}^{+}$ from states *i* to *j* is

5 $$f_{ij}^{+}=\pi_{i}\cdot q_{i}^{-}\cdot K_{ij}\cdot q_{j}^{+}$$

where π_*i*_ is the equilibrium population of state i, and $q_{i}^{-}$ $\left(q_{j}^{+}\right)$ is the backward (forward) committor. Partitioning states into source *A*, target *B*, and intermediates *I*, $q_{i}^{+}$ is the probability of reaching *B* before *A* from *i*, with $q_{i}^{+}=0$ when *i* ∈ *A*, $q_{i}^{+}=1$ when *i* ∈ *B*, and

6 $$q_{i}^{+}=\underset{j}{\sum }K_{ij}q_{j}^{+},i\in I$$

Likewise, $q_{i}^{-}$ is the probability of having come from *A* rather than *B*, with $q_{i}^{-}=1$ when *i* ∈ *A* and $q_{i}^{-}=0$ when *i* ∈ *B*. For a possibly non-reversible process in SEI evolution, it is obtained from the same equation on the time-reversed kernel $K_{ij}^{\mathrm{rev}}=\pi_{j}K_{ji}/\pi_{i}$ . This flux quantifies productive lithium transport between distinct local environments in the SEI and enables identification of dominant pathways and kinetic bottlenecks.

### Case Study in LPSCl/Li System

We first applied GET-SEI to characterize lithium dynamics in the LPSCl/Li interphase. Experimentally verified SEI thicknesses span hundreds of nanometers.^15,20^ To model nanoscale SEI formation, we employed neural-network potential (NNP) and performed neural-network molecular dynamics (NNMD) simulations (Supplementary Table 1). The LPSCl/Li interface model was built from the (100) surface of LPSCl and (001) surface of Li metal with a total of 2960 atoms. A 1.0 ns NNMD simulation was then carried out in the NVT ensemble at 300 K to sample interphase evolution (Figure 2a). Li-centered local-environment graphs were subsequently constructed from the NNMD trajectories (Methods section).

**Figure 2:**
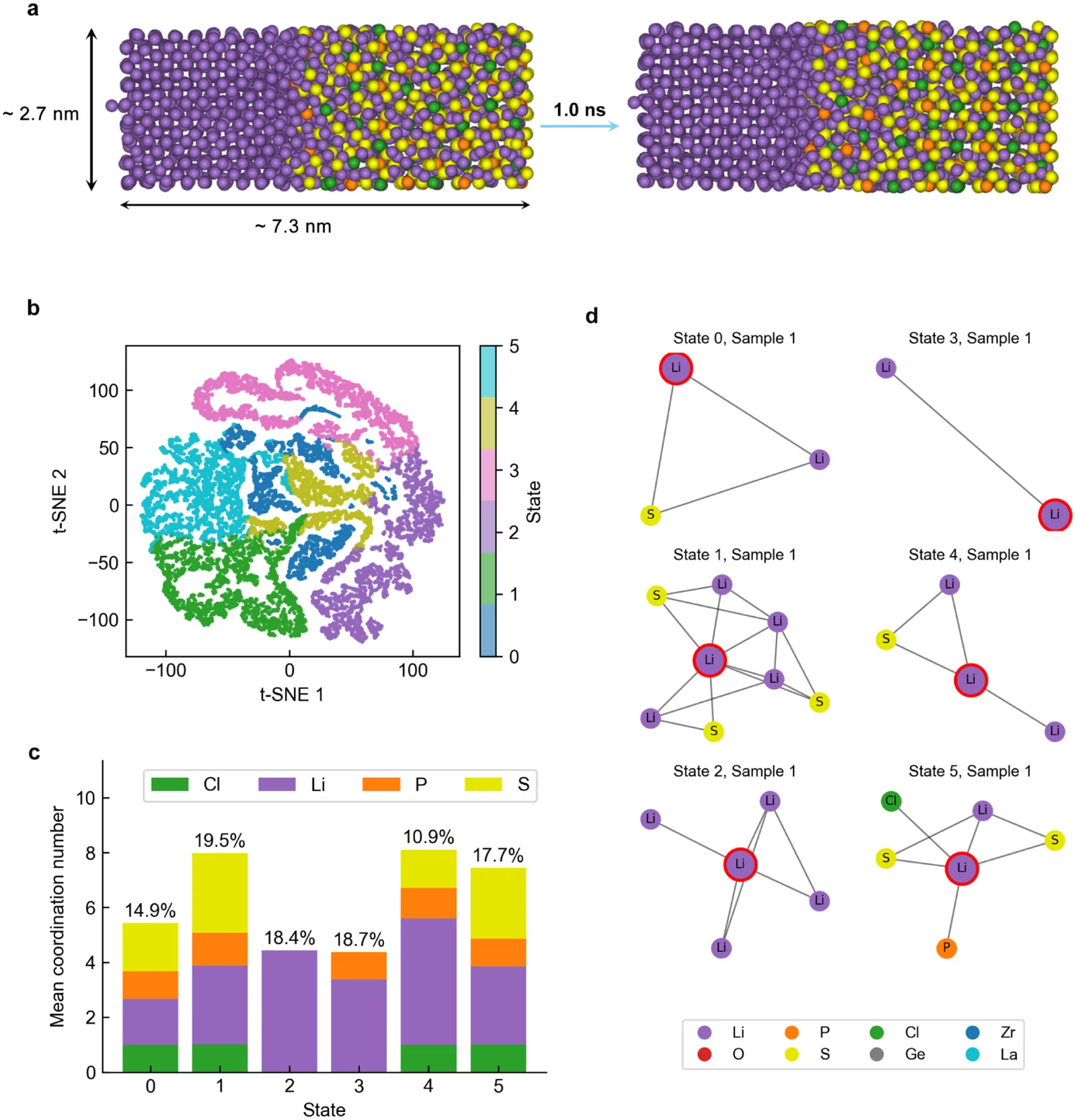
Representative local environments identified through graph contrastive learning in LPSCl/Li. **a,** LPSCl/Li model used for molecular dynamics simulation before and after 1.0 ns. **b**, t-SNE visualization of the embedded space in 2 dimensions. **c**, Mean coordination number of six states (S0–S5) in LPSCl/Li under a 4.0 Å cutoff. **d**, Representative graphs of six states (S0–S5) in LPSCl/Li system. The center lithium is denoted with a red circle.

To obtain robust local-environment classification while avoiding both over-fragmented and overly coarse observables, we selected a six-state decomposition (S0–S5), which yielded a Silhouette score of 0.29 (Figure 2b, Supplementary Table 2, Supplementary Note 1) These states exhibit distinct coordination environments and represent dominant structural motifs during interphase evolution. (Figure 2c–2d) Additional representative samples in the embedding space are provided in Supplementary Figure 1. We then linearized the lithium dynamics in the original chemical space using the Koopman operator K with a lag time of 80.0 ps, identifying five dominant dynamical processes in the LPSCl/Li SEI system (Figure 3a).

**Figure 3:**
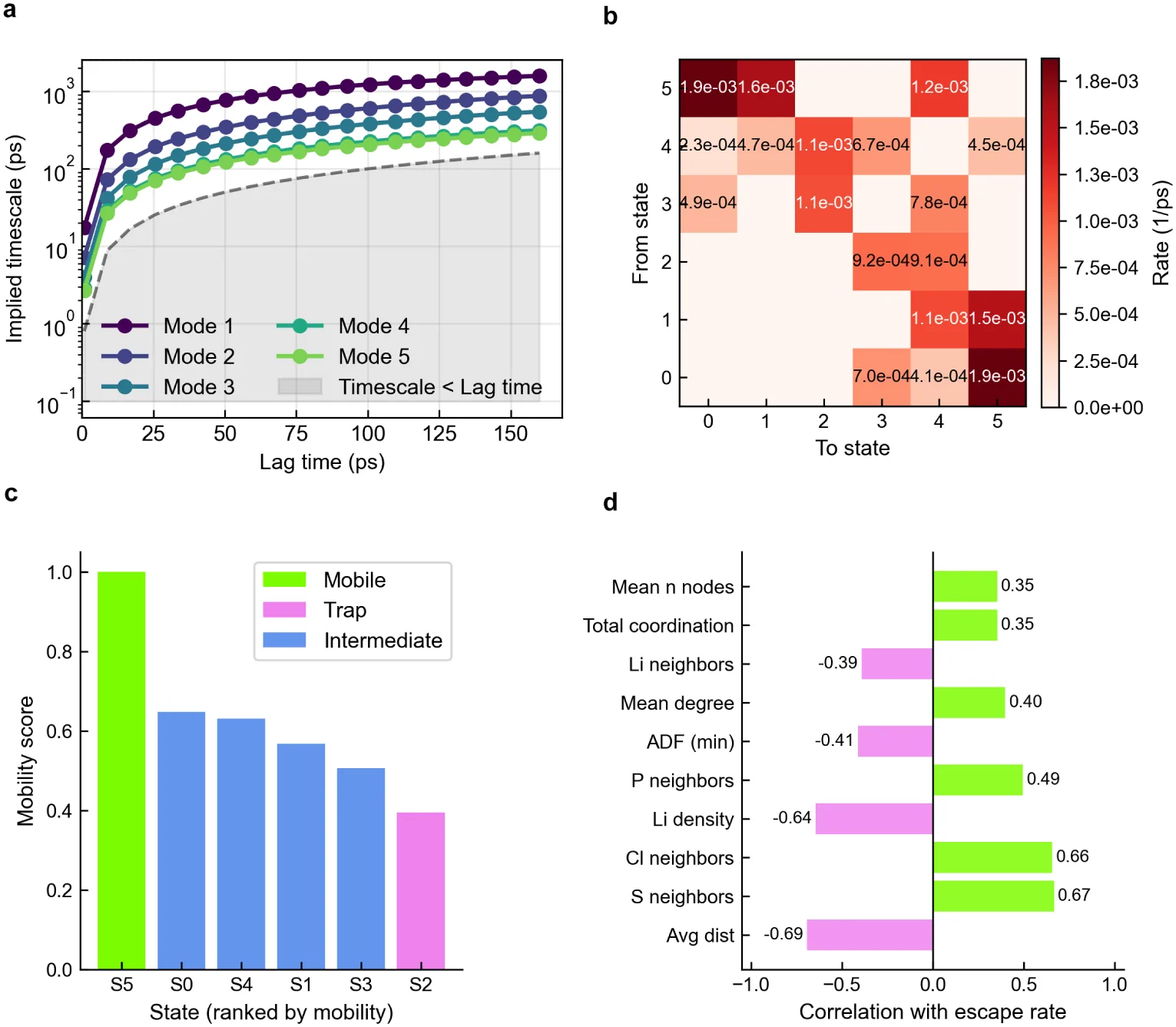
Lithium mobility analysis in LPSCl/Li. **a,** Implied time scale plot derived from the Koopman process. The dashed gray line denotes lag time equals relaxation time scale. **b**, Continuous-time rate matrix distribution for S0–S5 lithium states. Diagonal entries representing the negative escape rates are omitted from the heatmap. **c**, Mobility ranking of S0–S5 states in LPSCl/Li. **d**, Correlation between escape rates and node features of lithium graphs.

The continuous-time rate matrix (Figure 3b) quantifies lithium transfer kinetics among states. State S5 shows the highest migration propensity, consistent with its coordination environment resembling bulk LPSCl, and exhibits rapid transition rates to S0 (1.9 × 10^–3^ ps^–1^) and S1 (1.6 × 10^–3^ ps^–1^). Notably, S4, characterized by high lithium density and weak single-anion (S/P) coordination, (Figure 3d, Supplementary Figure 1) enables reversible transitions to both the solid-electrolyte-like region (S5) and lithium-anode-like region (S2). As shown in Figure 3c, the mobility score (normalized escape rate) identifies S2 as the kinetic bottleneck state which has highest lithium density, with the overall mobility ranking: S5 > S0 > S4 > S1 > S3 > S2. Correlation analysis between graph-derived local features and escape rates further indicates that larger average Li-neighbor distances and higher local Li density hinder fast transport, whereas the presence of anion-rich coordination (S/Cl) promotes lithium escape to other states (Figure 3d).

The escape rate matrix ***R*** captures global transitions among local SEI environments, but it does not fully resolve the detailed mechanisms of lithium migration. To uncover pathway-specific transport, we quantified reactive fluxes using TPT.^44,45^ As shown in Figure 4a, the equilibrium flux *f_ij_*, which represents balanced total flow independent of source-target directionality, is highest among the lithium-rich states S2, S3, and S4, consistent with active lithiation/delithiation processes in the SEI region. We then filtered *f_ij_* to extract transitions that originate from a specified source ***A*** and terminate in a target set ***B***, yielding the reactive flux $f_{ij}^{+}$ (Figure 4b–4c, eq 5). Based on these reactive fluxes, we constructed the state-transition network (Figure 4c). In pairwise analysis, the dominant reactive channels are S2 → S4 (associated with Li–S/P bond formation) and S2 → S3 (delithiation), confirming strong interfacial coupling between LPSCl and the lithium metal region.

**Figure 4:**
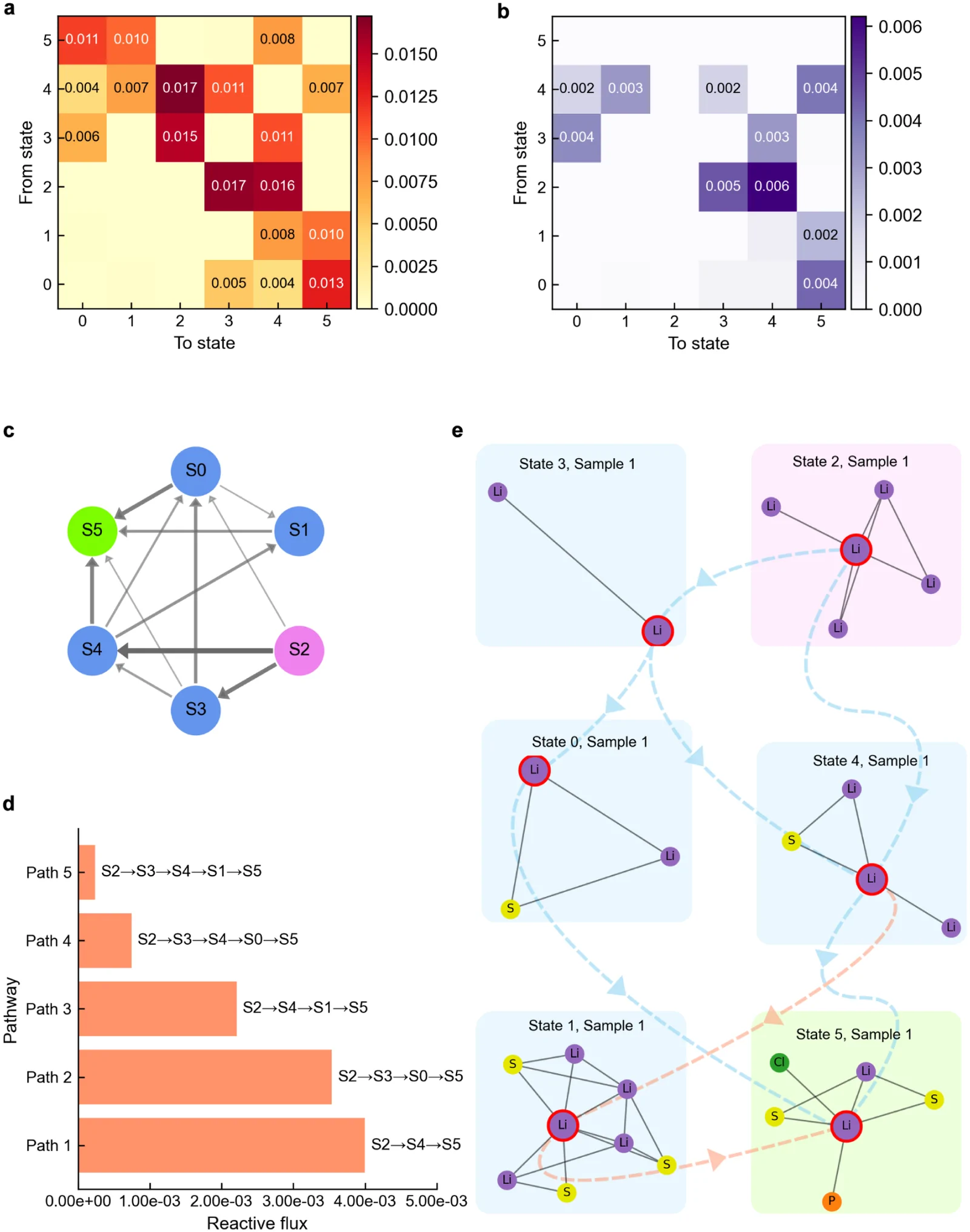
Transition pathways of lithium ions in LPSCl/Li. **a–b**, Equilibrium and reactive flux between different states in the LPSCl/Li SEI. **c**, Transition network of lithium states (S0–S5). The initial and final states are colored pink and green, respectively. The width of lines represents the relative strength of the reactive flux. **d**, Top five lithium conduction pathways from S2 to S5 in LPSCl/Li SEI. **e**, Illustration of different pathways in the LPSCl/Li SEI. Dashed blue and orange lines represent the unrestricted and restricted paths, respectively. The initial, intermediate, and final states are denoted with a pink, blue, and green background, respectively.

Conduction routes and their associated reactive fluxes between any two states were further evaluated using a modified Dijkstra algorithm that identifies minimum edge-weight pathways (Methods). As shown in Figure 4d, transitions from the least mobile state (S2) to the most mobile state (S5) proceed through two principal routes: (i) Path 1: S2 → S4 → S5 (dominant), and (ii) Path 2: S2 → S3 → S0 → S5 (secondary). These pathways suggest a common migration pattern: Li first enters a lower Li-density environment (optionally S3), is transiently coordinated by anion-rich environments (S0 or S4), and then migrates into the solid-electrolyte-like state (S5). In bottleneck routes such as Path 5: S2 → S3 → S4 → S1 → S5, the S4 → S1 transition is rate-limiting. This barrier is associated with stronger multi-anion coordination of Li in high-Li-density state S1, which suppresses onward transport. These results suggest that SEI design should minimize formation of such trapping states through composition and local-structure control.^6^

### Case Studies in Other SEI Systems

Beyond the LPSCl/Li interphase, we generalized GET-SEI to two additional interphase systems, LGPS/Li and LLZO/Li, to evaluate cross-system transferability. These systems exhibit fundamentally different interphase structures due to distinct solid-electrolyte/lithium–metal interaction. In LGPS/Li, relatively soft interfacial contact promotes formation of an intermediate SEI region spanning nanometer length scales^48,49^ (Figure 5a). In contrast, LLZO/Li shows weak interfacial reactivity with lithium metal and forms a comparatively sharp interface without a clearly resolved intermediate SEI layer^50,51^ (Figure 5b). For both systems, dominant local environments were classified into six states (S0–S5), consistent with the LPSCl/Li setup for direct comparison (Figure 5c–d, Supplementary Figure 2–3), with measured silhouette scores >0.29 (Supplementary Table 2).

**Figure 5:**
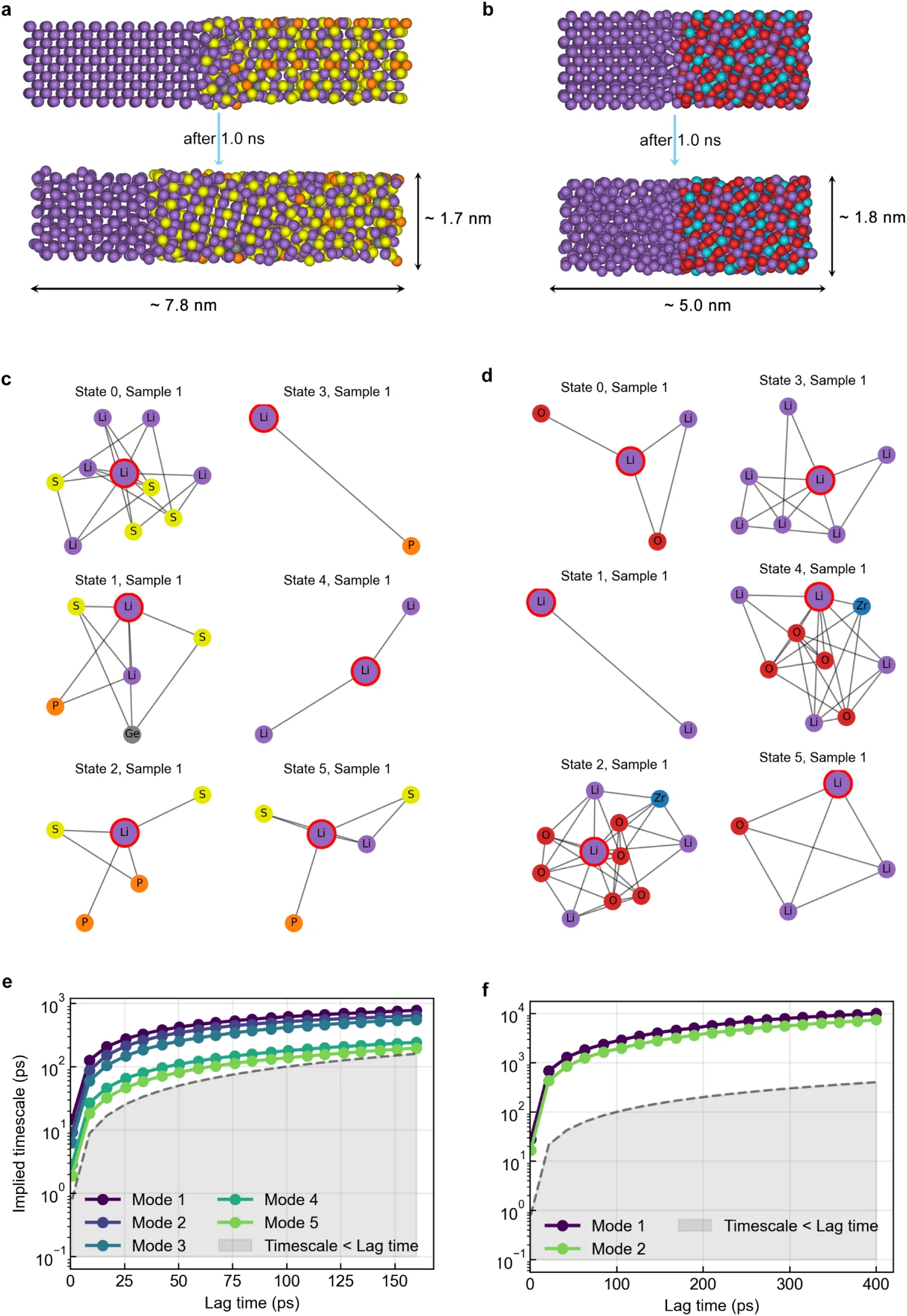
Local environments and dynamics in LGPS/Li and LLZO/Li. **a–b,** Interface models of LGPS/Li and LLZO/Li employed in molecular dynamics simulations, respectively. **b–c**, Representative graphs of classified six states in LGPS/Li and LLZO/Li SEIs, respectively. The center lithium is denoted with a red circle. **e–f**, Implied time scale plot derived from the Koopman process in LGPS/Li and LLZO/Li SEIs, respectively. The dashed gray line denotes lag time equals relaxation time scale.

Reconstruction of interphase dynamics using Koopman analysis reveals markedly different lithium-transport behavior in LGPS/Li and LLZO/Li. In LGPS/Li, larger average Li-neighbor distances and higher local Li density in the SEI region correlate with lower escape rates. (Supplementary Figure 4) In contrast, for LLZO/Li, both increased Li density and larger average distance correlate with higher escape rates (Supplementary Figure 5). This opposite trend reflects fundamentally different local-environment landscapes (Figure 5c–5d): In LGPS/Li, the S5 (the most mobile state; Supplementary Figure 6) is characterized by low Li density with anion coordination (e.g., P/S), which facilitates transitions between Li-rich anode regions and anion-rich LGPS environments. In LLZO/Li, mobile states are instead Li-rich with low oxygen content (S5) or nearly pure Li environments (S3/S1), consistent with stronger oxygen-associated trapping effects. Notably, S2 (with the lowest mobility score; Supplementary Figure 7) in LLZO/Li is oxygen-rich and acts as a kinetically inert barrier that suppresses lithium conduction. Koopman mode analysis further resolves five dynamical processes in LGPS/Li but only two in LLZO/Li (Figure 5e–5f), reinforcing the more limited state-to-state transitions in LLZO/Li.

Reactive lithium flux analysis further confirms the distinct lithium-transport mechanisms in these systems. As a representative comparison, we consider transitions from the least mobile state to the most mobile state (LGPS/Li: S4 → S5, LLZO/Li: S2 → S5). In LGPS/Li, transport proceeds through multiple pathways, with the direct S4 → S5 channel carrying the dominant reactive lithium flux (Figure 6a–6b). Pathways involving strongly anion-coordinated intermediates (e.g., S2), such as S4 → S3 → S2 → S5, substantially suppressed to approximately one-third of the direct flux, indicating kinetic penalties in these environments. The presence of these diversified pathways explains the superior Li mobility in the LGPS based SEI.^48,49^ In LLZO/Li, by contrast, conduction is dominated by a single principal route from S2 to S5, corresponding to migration from oxygen-rich to lithium-rich environments (Figure 6c–6d). Alternative pathways (Paths 3–5) that pass through intermediate states (e.g., S4, S0, and S1) with progressively reduced oxygen coordination are nearly blocked, supporting the conclusion that oxygen-rich environments in LLZO/Li stabilize a kinetically inert interphase region.^50,51^

**Figure 6:**
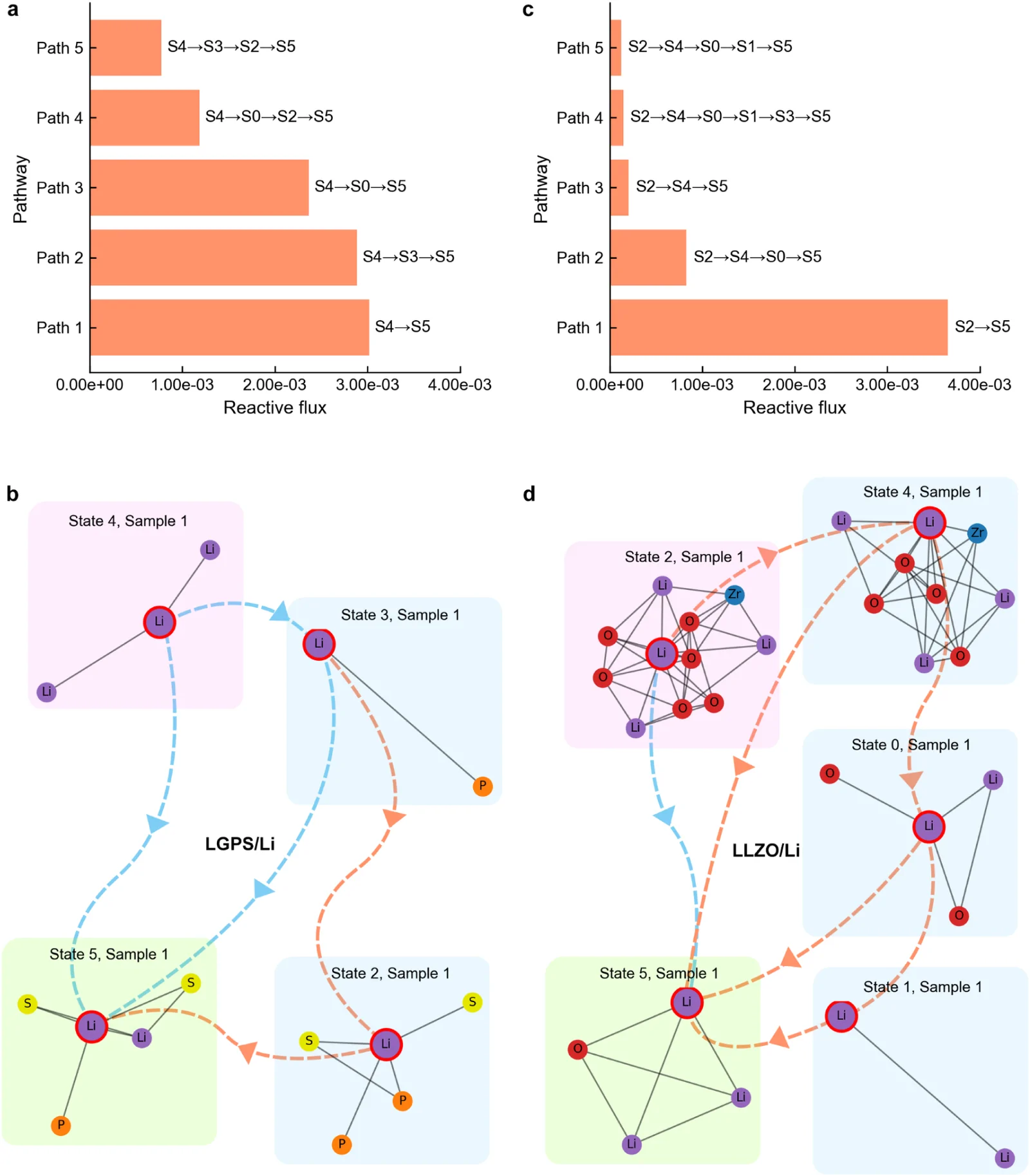
Transition pathways of lithium ions in different SEIs. **a–b**, Reactive fluxes and illustration of top five dominant lithium transport pathways (S4 → S5) in LGPS/Li, respectively. **c–d**, Reactive fluxes and illustration of top five dominant lithium transport pathways (S2 → S5) in LLZO/Li SEI. Dashed blue and orange lines represent the unrestricted and restricted paths, respectively. The center lithium is denoted with a red circle. The initial, intermediate, and final states are denoted with a pink, blue, and green background, respectively.

Taking together, these cross-system comparisons demonstrate that GET-SEI not only resolves local lithium-environment states, but also links them quantitatively to kinetic transitions and dominant transport pathways. By unifying GCL-based state discovery with EDMD and TPT flux analysis, the framework reveals how chemistry-specific local environments govern mobility, bottlenecks, and pathway diversity across sulfide and oxide interphases. These results establish a mechanistically interpretable basis for comparing interphase transport in different SEIs and provide actionable descriptors for interphase optimization.

### Validation of GET-SEI Kinetic Observables against MD Transport Metrics

The mobility score and lithium fluxes obtained from GET-SEI provide a quantitative measure of lithium kinetics in each SEI system. To further assess the robustness of the framework, we examine their correlation with transport observables that can be computed directly from MD trajectories.

System-level lithium-ion activity can be quantified by summing the reactive fluxes between all pairs of states. Because both larger fluxes and greater lithium displacements indicate higher activity, the total lithium flux is expected to correlate with mean square displacement (MSD). We therefore compare the system-level reactive flux with the whole-system Li MSD evaluated at a lag time of 950 ps (Supplementary Figure 8). This comparison links active-site transfer identified by GET-SEI to physical displacements accessible from raw MD data. The total pairwise reactive flux *f_tot_* is

7 $$f_{tot}=\underset{i}{\sum }\underset{i\neq j}{\sum }f_{ij}^{+}$$

where $f_{ij}^{+}$ is lithium flux for a singleton source state *i* and target state j . The MSD is calculated through:

$$MSD=\frac{1}{N_{\mathrm{Li}}}\overset{i=1}{\underset{N_{\mathrm{Li}}}{\sum }}{|\Delta r_{i}\left(t_{0},\tau \right)|}^{2}$$ 8

where $N_{\mathrm{Li}}$ is the number of lithium atoms, and Δ*r_i_* (*t*_0_, τ) is the displacement between time *t*_0_ and time *t*_0_ + τ. Across LLZO/Li, LPSCl/Li, and LGPS/Li, the GET-SEI flux ranking matches the MSD ranking exactly (Spearman score *ρ_flux_* = 1.00), with a positive numerical relationship (Pearson score γ_flux_ = 0.71; Supplementary Table 3; Supplementary Note 2). Although limited to three systems, this comparison shows that *f_tot_* is consistent with directly measurable MSD.

The mobility score of each state quantifies the escape propensity of lithium within that state. To assess its validity, we compare it with the residence diffusion coefficient *D*_res_ defined from the net Li displacement accumulated during continuous same-state residence events (see Methods; Supplementary Table 4). In LLZO/Li, states ranked as more mobile by GET-SEI also exhibit larger displacement per unit time while remaining in that state (Spearman score *ρ*_mob_ = 0.83; Supplementary Note 3; Supplementary Figure 9), indicating that escape-rate-based mobility aligns with intra-state physical motion. In LPSCl/Li and LGPS/Li, the correlations are negative (*ρ*_mob_= −0.71 and −0.60, respectively; Supplementary Figures 10 and 11), indicating that high escape propensity need not coincide with large within-state diffusion coefficient; transport is instead mediated primarily by transitions between local environments, consistent with the more diversified pathways in Figures 4d and Figure 6a. Thus, *D*_res_ measures physical displacement during state residence, whereas escape-rate-based mobility captures the kinetic propensity to leave a state. Their system-dependent correlation distinguishes regimes of intra-state motion from those dominated by inter-state hopping, further supporting mobility analysis within the GET-SEI framework.

## Conclusion

Different SSEs interact with lithium metal anodes in distinct ways, leading to complex interfacial chemistries and structures. Relative to crystalline inorganic SSE phases, amorphous SEIs exhibit richer local-environment diversity and therefore more complex lithium dynamics. Quantitative characterization of lithium transport in these SEI regions is highly challenging but essential for rational optimization of all-solid-state battery performance. In this work, we develop GET-SEI, a general framework that uses self-supervised GCL to resolve diverse Li-centered local environments, then combines EDMD-based Koopman analysis and TPT to reconstruct nonlinear dynamics and quantify transport pathways in an interpretable way.

Applied to sulfide-based (e.g., LPSCl/Li and LGPS/Li) and oxide-based (e.g., LLZO/Li) interphases, GET-SEI quantitatively maps lithium mobility across distinct local states. Sulfide-based SEIs, especially LGPS/Li, exhibit multiple conductive pathways with higher pathway diversity, whereas LLZO/Li is dominated by oxygen-rich coordination environments that create kinetically inert barriers and suppress transitions. These results suggest a practical design principle for fast interfacial transport: increasing the connectivity and population of high-mobility states while suppressing strongly coordinated trapping states. More broadly, GET-SEI provides a transferable, mechanism-informed metric framework for evaluating and designing SEIs toward fast, stable lithium transport in all-solid-state batteries.

## Methods

### Neural-Network Potential Preparation

All the neural-network potentials (NNPs) were developed using MACE foundation model.^52^ Initial data was prepared through *ab initio* molecular dynamics (AIMD) simulations using CP2K.^53^ The Perdew–Burke–Ernzerhof (PBE) generalized gradient approximation (GGA)^54^ with the double-zeta valence polarized basis set and Goedecker–Teter–Hutter pseudopotentials were adopted.^55^ The auxiliary plane-wave basis was truncated at a density cutoff of 500 Ry, and van der Waals interactions were treated with the Grimme D3 correction. A Nosé-Hoover thermostat and Martyna-Tobias-Klein barostat were used, each with a coupling constant of 1.0 ps. The time step was 2.0 fs.

For the LPSCl/Li system, six initial structures containing Li_2_S, LiCl, and Li_3_P units were generated with PACKMOL^56^ based on experimental findings,^15^ together with LPSCl^57^ and an explicit Li/LPSCl interfacial model. Each system was simulated by 10.0 ps of AIMD at 300–1500 K and 1 atm in the NPT ensemble. Two frames from each NPT trajectory were then selected for follow-up NVT simulation with multithermal sampling using on-the-fly probability method.^58^ The final dataset comprised 28648 training configurations and 7162 test configurations. The final NNP was obtained by further fine-tuning the mace-mpa-0-medium model.^52^ Four trained models were evaluated, with accuracy metrics reported in Supplementary Table 1. For other SEI systems (LGPS/Li and LLZO/Li), the mace-mpa-0-medium model was directly employed for simulations.

### Molecular Dynamics Simulation

All NNMD trajectories were generated through the ASE package.^59^ A timestep of 2.0 fs was used for all simulations. All NNMDs employed a 2.0 fs timestep. For each system, a 1.0 ns NNMD simulation under NVT ensemble at 300 K was performed with a Langevin thermostat (coupling constant: 1.0 ps). Cell parameters and atom counts are summarized below: (1) LPSCl/Li: 27.04 × 27.92 × 95.22 Å, including 2960 atoms. (1) LGPS/Li: 17.38 × 17.20 × 96.95 Å, including 1200 atoms. (3) LLZO/Li: 18.47 × 18.48 × 71.79 Å, including 1167 atoms.

### Graph Construction

Individual lithium graphs were created through Pytorch geometric.^60^ The graph edges were constructed through cutoff-based algorithm using a cutoff of 3.0 Å. Four kinds of node features were selected: (1) Atomic number, (2) Average distance between the center lithium atom and neighbor nodes, (3) Number of each element in the local coordination shell (a larger shell radius of 4.0 Å is used), (4) Summary of statistics of angular distribution functions (ADFs) were used to describe the positional distribution of the atoms, including values of mean, standard deviation, minimum, maximum, and median of the ADFs. The ADFs were calculated by computing angles for all unique pairs of neighbors within the cutoff distance of 4.0 Å.

### Graph Contrastive Learning Architecture

The input of the GCL is lithium graphs generated from NNMD trajectories. 5% of the NNMD frames were used as training data for the local environment classification. For the augmented views of Li graphs, the 0.1 edges were randomly dropped out, and 0.2 node features were randomly masked. The temperature σ was selected as 0.5 for a good balance between the sharp and smooth embeddings. After data augmentation, they were fed into the graph attention network (GAT) encoder, which includes 2 layers with multi-head attention (4 heads), learning the neighbors surrounding the Lithium atom. Then the output was projected to a 32-dimensional embedding space. Then we clustered the embedding space through Gaussian mixture model through sci-kit learn package.^61^ Finally, we assigned states to each Li at each frame in our simulated trajectories.

### Construction of Koopman Operator through EDMD

EDMD approximates the Koopman operator using a dictionary of basis functions $\left\{\psi_{1},\psi_{2},\cdot \cdot \cdot ,\psi_{D}\right\}$ . Common dictionaries include monomials, radial basis functions and through neural networks like VAMPnets.^27,29^ Here, since we already have discrete states for lithium atoms after graph contrastive learning, we use the indicator functions through one-hot encoding which is efficient and simple:

9 $$\psi_{i}\left(x\right)=\{\begin{array}{ll} 1, & x=i\\ 0, & x\neq i \end{array}$$

Then we collected the data pairs (*x*_*n*_, *x*_*n*+1_) from the NNMD trajectories. These data pairs were transformed into an observable space (*g*(*x*_*n*_), *g*(*x_n_*_+1_)), with each expressed by the dictionary functions *ψ*:

10 $$g\left(x_{n}\right)=\left[\psi_{1}\left(x_{n}\right),\psi_{2}\left(x_{n}\right),\cdot \cdot \cdot ,\psi_{D}\left(x_{n}\right)\right]$$

Through solving the least squares problem in the observable space, we could have the finite-dimensional matrix approximation ***K*** of K :

$$K\approx K={\left(G_{n}^{T}G_{n}\right)}^{-1}G_{n}^{T}G_{n+1}$$ 11

where ***G**_n_* and ***G**_n_*_+1_ are matrices form of *g*(*x_n_*) and *g*(*x_n_*_+1_), whose rows are the lifted observables at time *n* and *n* + 1 respectively.

### Algorithm in Finding Dominant Pathways

The dominant pathways between source and target states were identified using TPT combined with a greedy flux decomposition. After having the reactive fluxes $f_{ij}^{+}$ (eq 5), we decomposed it into individual pathways using an iterative algorithm: at each iteration, a modified Dijkstra search finds the path from source to target with the maximum bottleneck flux, that path’s bottleneck flux is subtracted from all its edges, and the procedure repeats on the residual flux network until the desired number of pathways is extracted. Finally, we have the results of an ordered set of dominant pathways ranked by their flux contributions, providing a mechanistic decomposition of how reactive transitions flow through the intermediate states of the SEI system.

### Calculation of Residence Diffusion Coefficient

For tracked Li ion, a residence event occupies state *s* at *t*_start_, and a state change at *t*_end_. The residence duration is

12 $$\tau_{\mathrm{res}}=\left(t_{\mathrm{end}}-t_{\mathrm{start}}\right)\Delta t$$

Here τ_res_ is the residence event duration, Δ*t* is the time interval between each frame. Using lithium-ion positions **r**_i_(*t*), the net displacement over the residence event is

13 $$\Delta r_{\mathrm{res}}=r_{i}\left(t_{\mathrm{end}}\right)-r_{i}\left(t_{\mathrm{start}}\right)$$

The event-level residence diffusion coefficient *D*_res_ is finally calculated in the form:

14 $$D_{\mathrm{res}}=\frac{{|\Delta r_{\mathrm{res}}|}^{2}}{2d\tau_{\mathrm{res}}}$$

where *d* is the dimensionality of the system. With units Å^2^/ps, *D*_res_ representing event-level physical diffusion coefficient for lithium ions in SEI systems. For each state *s*, the median *D*_res_ over all residence events in that state is used for comparison.

## Supplementary Material



## Data Availability

The MD trajectories of the LPSCl/Li, LGPS/Li, and LLZO/Li SEI systems are available at 10.5281/zenodo.22014813. The sample code to perform the analysis is available at https://github.com/DXiming/getsei.

## Notes

Additional information on representative graphs of LPSCl/Li, LGPS/Li and LLZO/Li SEI systems; details on mobility ranking and correlation of the escape rate with node features in LGPS/Li and LLZO/Li SEI systems; further details of neural-network potential training of LPSCl/Li system; analysis of lithium graph classification through a Gaussian mixture model in different SEI systems; details on correlation between residence diffusion coefficient and mobility score from GET-SEI (PDF)
