# Supplementary material for: Unraveling Lithium Dynamics in Solid Electrolyte Interphase: From Graph Contrastive Learning to Transport Pathways
Source: J Chem Theory Comput. 2026 Sep 9;22(18):9676–87. doi: 10.1021/acs.jctc.6c00987 (PMC13602929; doi:10.1021/acs.jctc.6c00987)
Supplement: Supplementary file 1 [file ct6c00987_si_001.pdf]

Supporting Information

Unraveling Lithium Dynamics in Solid Electrolyte Interphase: From Graph Contrastive Learning to Transport Pathways

Qiye Guan* and Yongqing Cai*

Institute of Applied Physics and Materials Engineering, University of Macau, Taipa, Macau 999078, China

**Corresponding author*

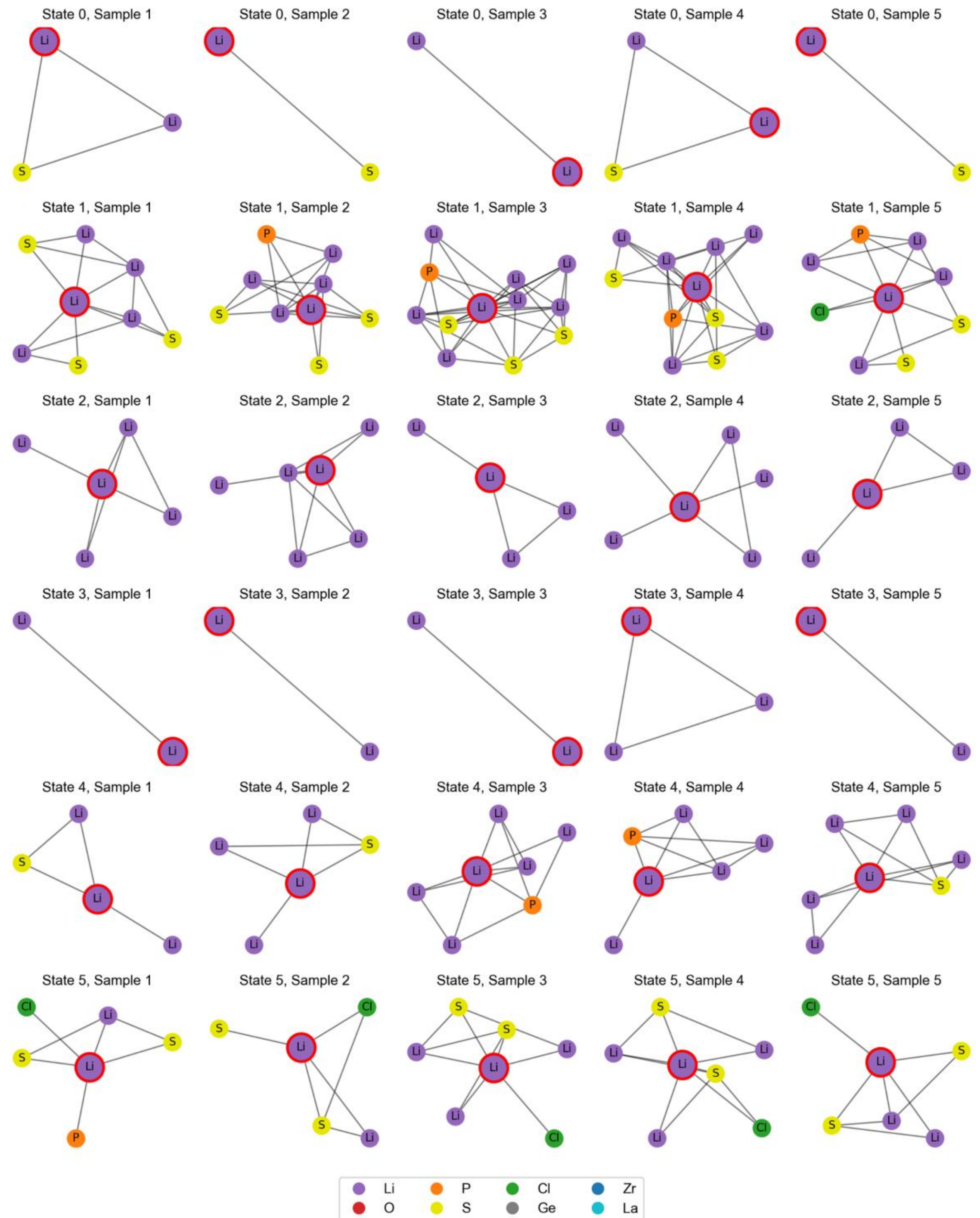

Supplementary Fig. 1 Five representative graphs of each classified state in LPSCl/Li.

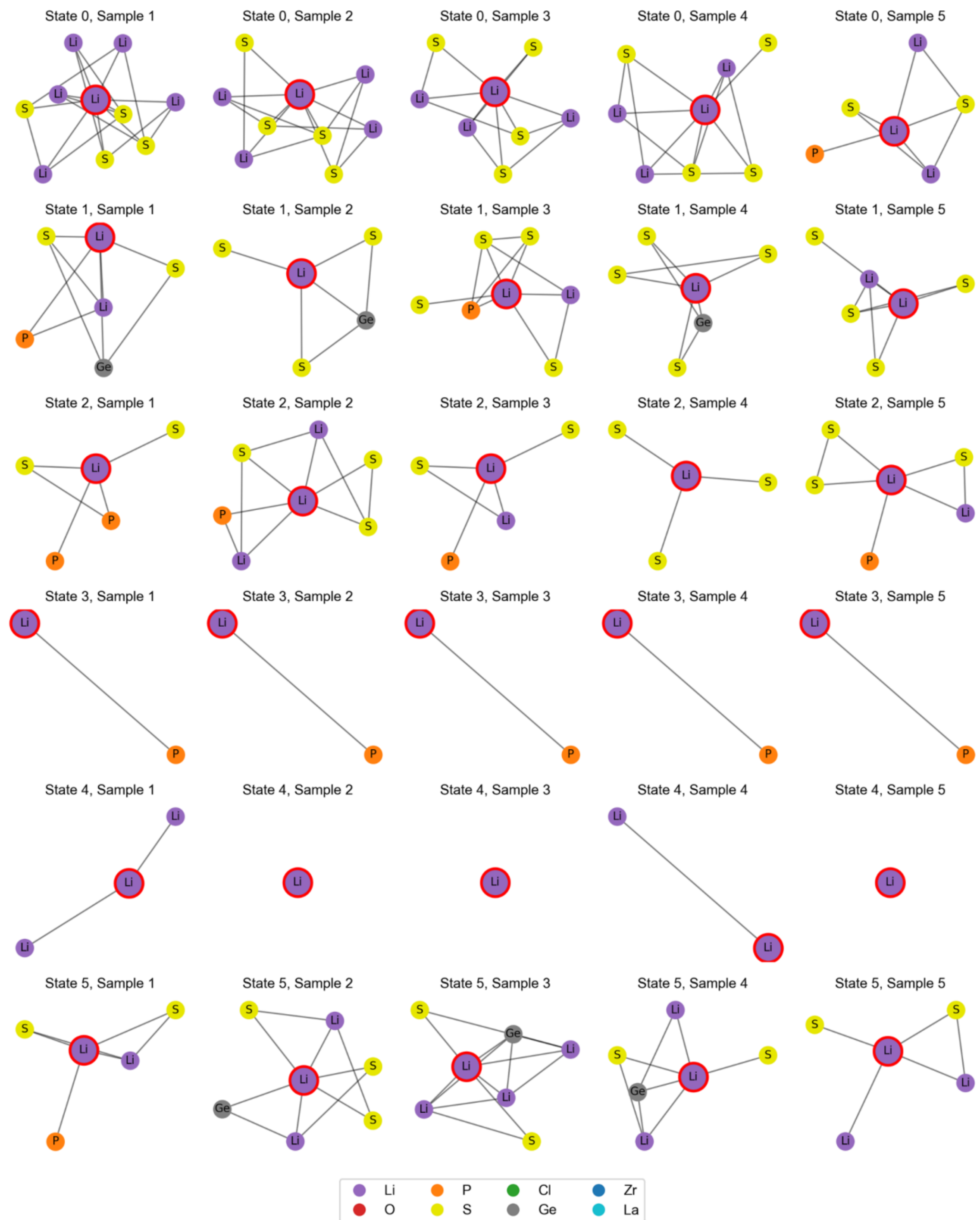

Supplementary Fig. 2 Five representative graphs of each classified state in LGPS/Li.

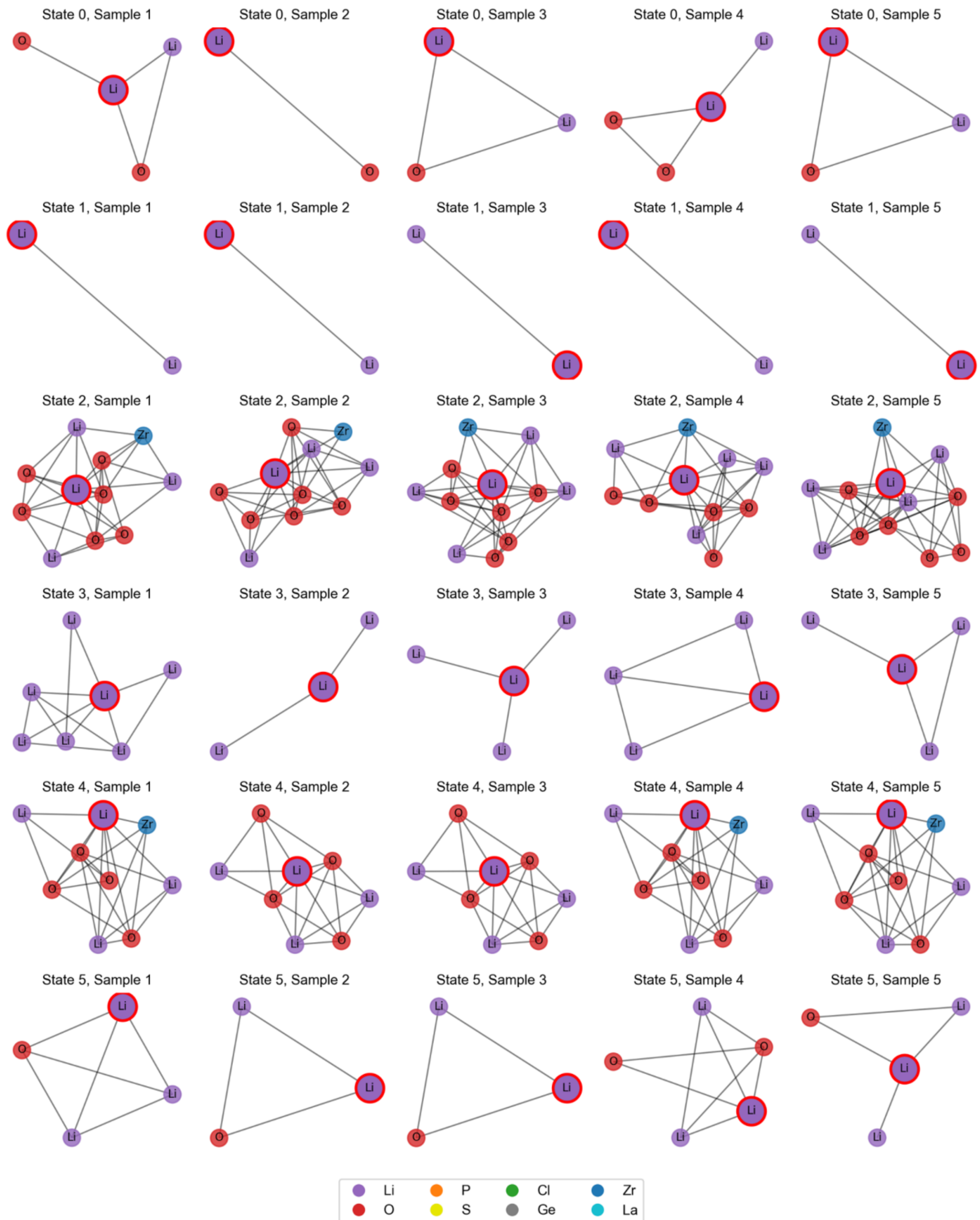

Supplementary Fig. 3 Five representative graphs of each classified state in LLZO/Li.

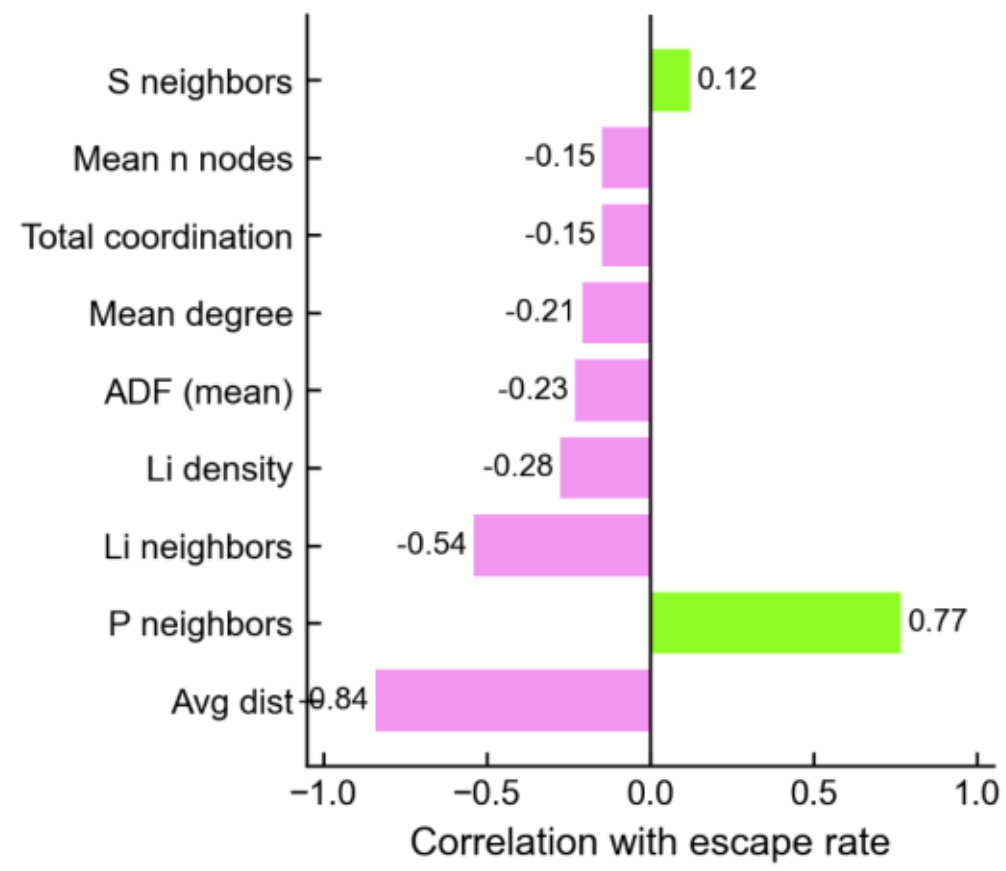

Supplementary Fig. 4 Correlation of escape rate with node features of Li graph in LGPS/Li.

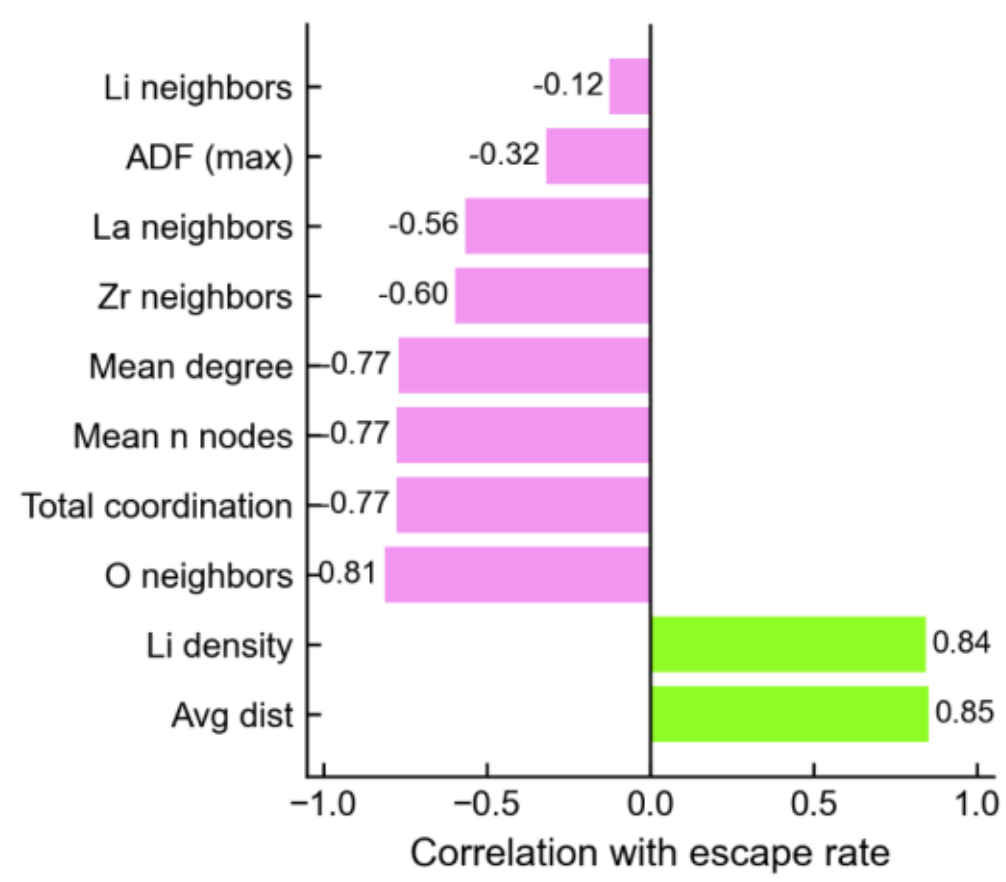

Supplementary Fig. 5 Correlation of the escape rate with node features of Li graph in LLZO/Li.

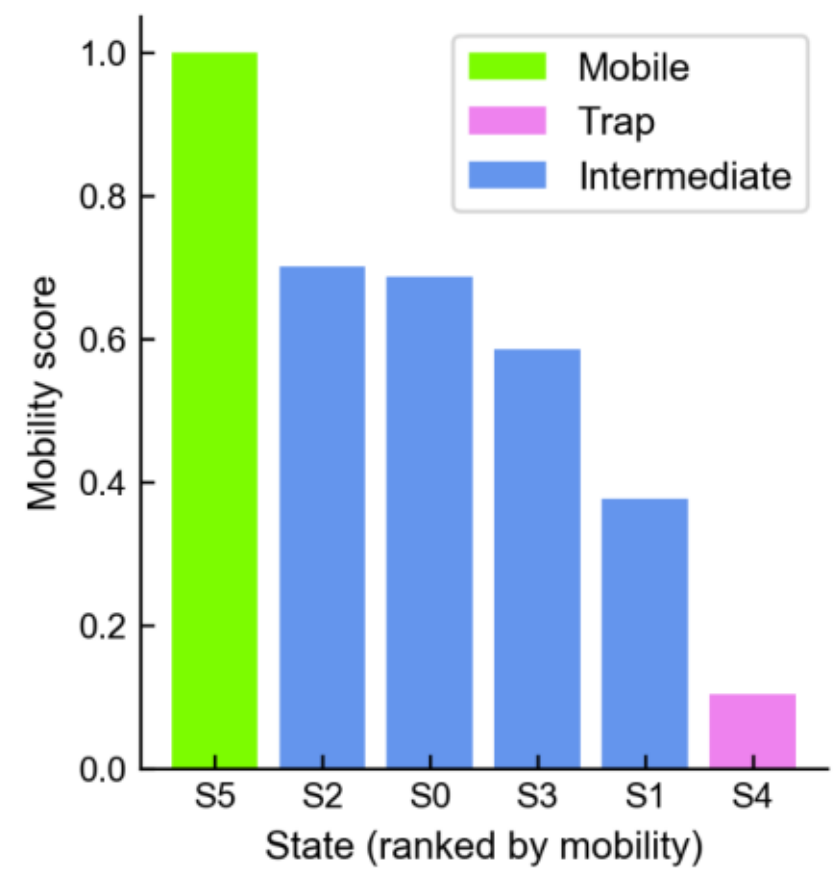

Supplementary Fig. 6 Mobility ranking of S0–S5 states in LGPS/Li.

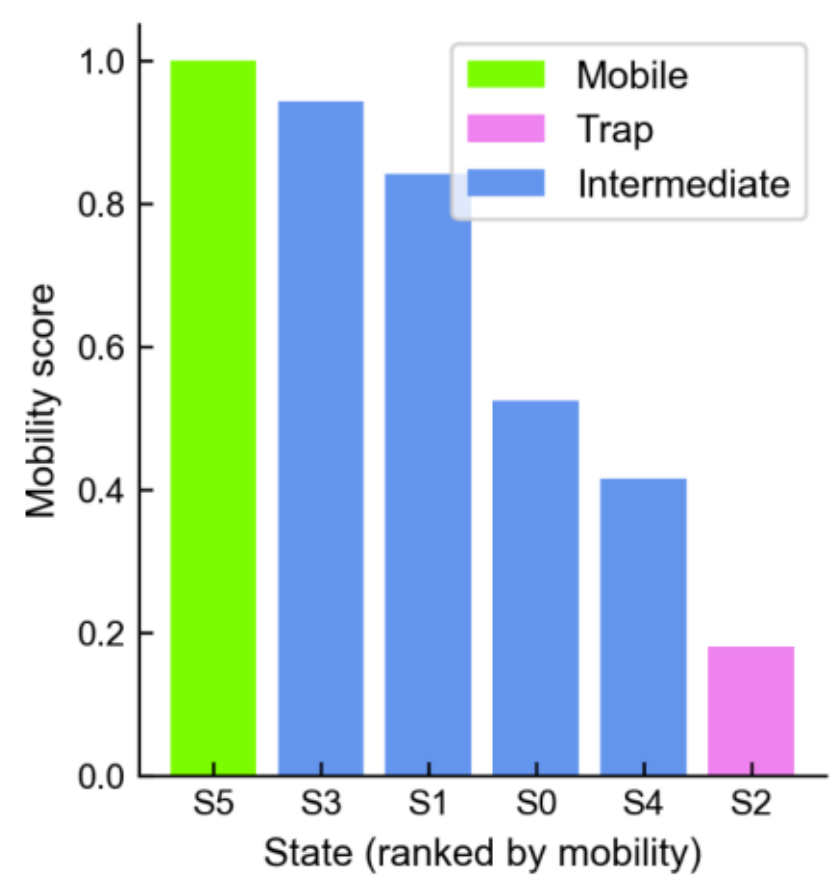

Supplementary Fig. 7 Mobility ranking of S0–S5 states in LLZO/Li.

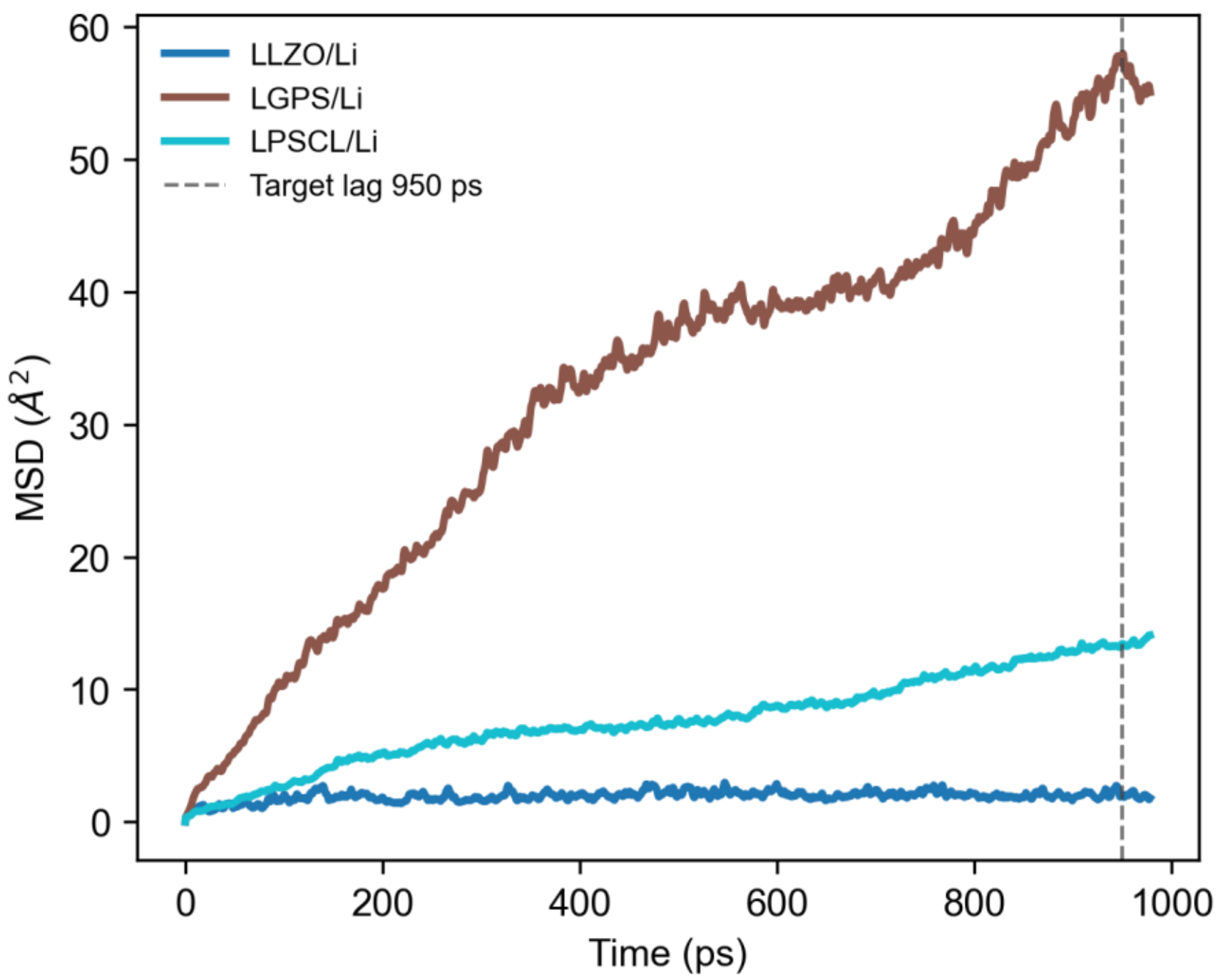

Supplementary Fig. 8 Mean square displacement (MSD) calculation of lithium atoms in different SEI systems.

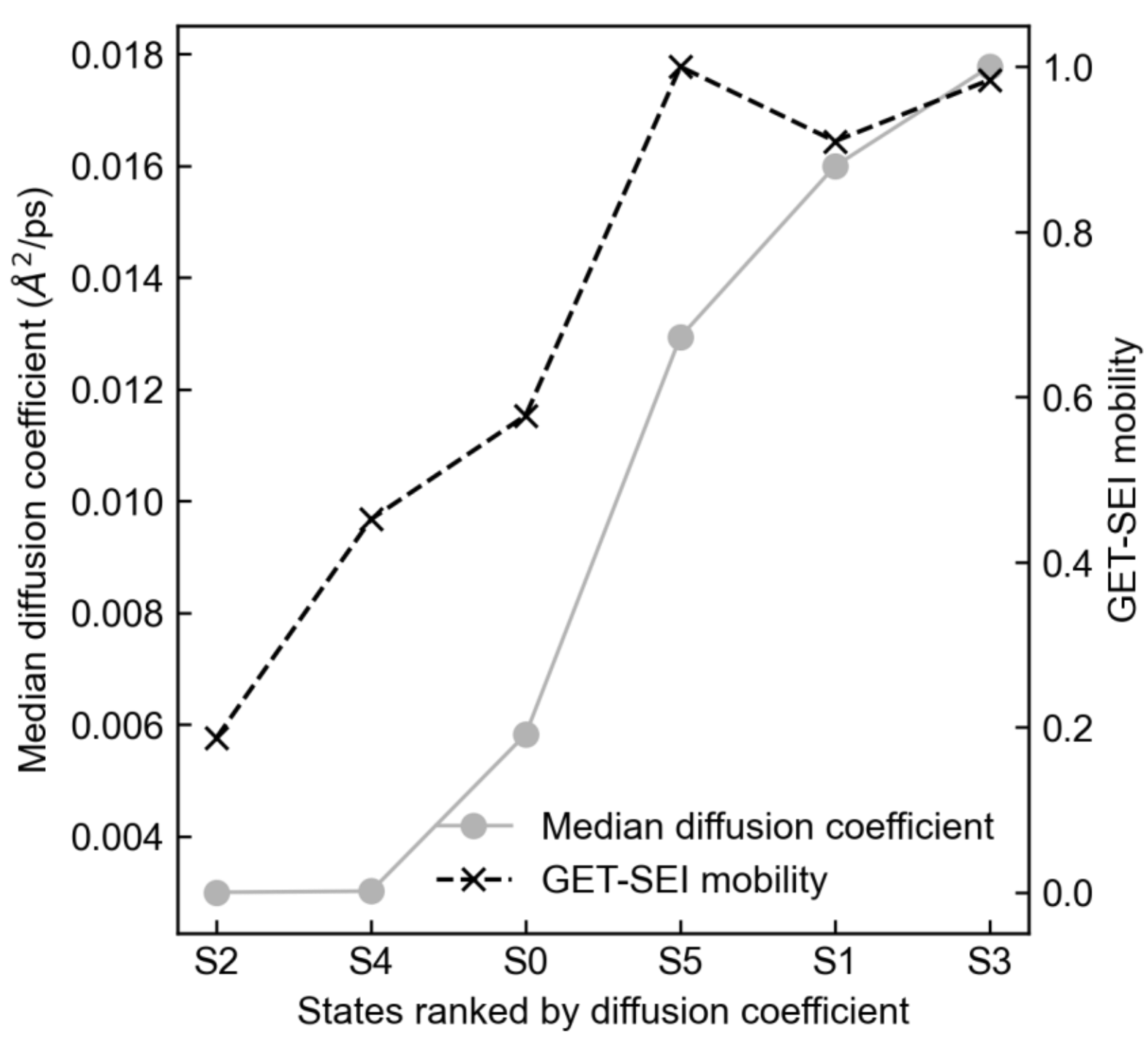

Supplementary Fig. 9 GET-SEI mobility and residence diffusion coefficient of all states in LLZO/Li.

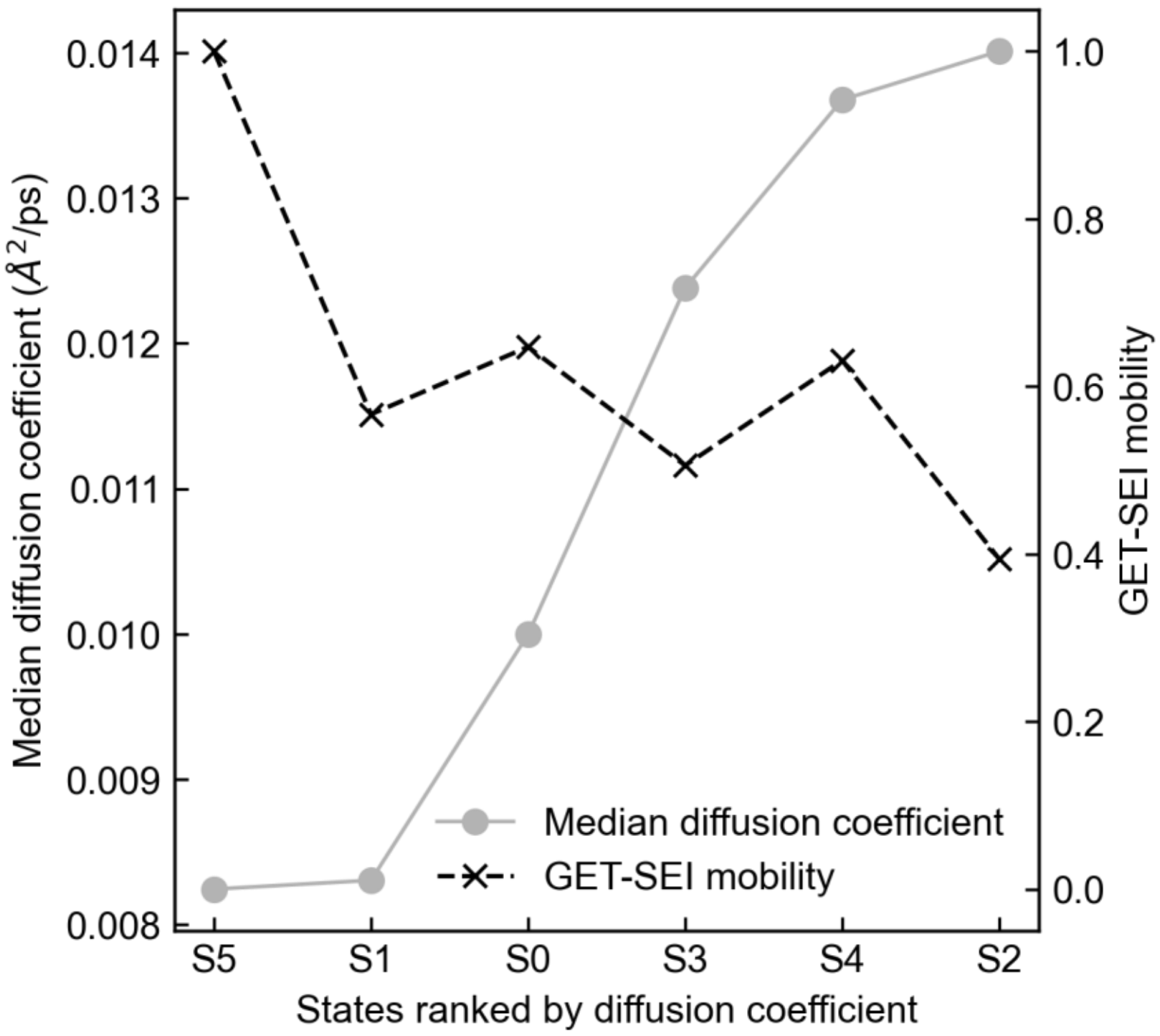

Supplementary Fig. 10 GET-SEI mobility and residence diffusion coefficient of all states in LPSCl/Li.

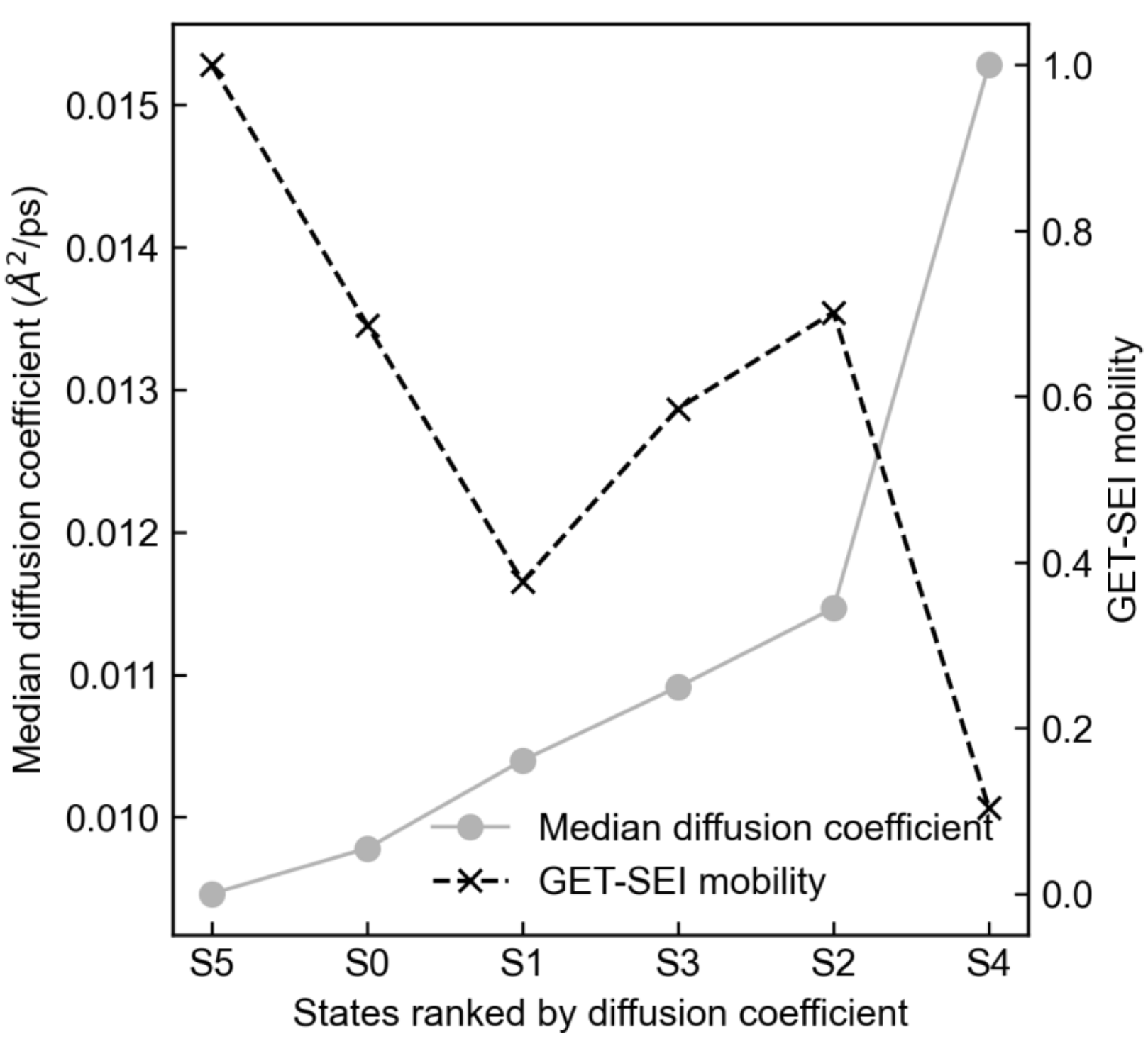

Supplementary Fig. 11 GET-SEI mobility and residence diffusion coefficient of all states in LGPS/Li.

Supplementary Table 1. Root mean square errors (RMSEs) of trained neural-network potentials (LPSCl/Li).

	Energy (meV/atom)	Force (meV/Å)
Model-1	0.6	21.4
Model-2	0.6	21.8
Model-3	0.6	21.2
Model-4	0.6	21.5

Supplementary Table 2. Silhouette score of classification through gaussian mixture model in different SEI systems.

	LPSCl/Li	LGPS/Li	LLZO/Li
Silhouette score	0.29	0.30	0.33

Supplementary Table 3. Mean square displacements (MSDs) and total reactive fluxes in different SEI systems.

	LLZO/Li	LPSCl/Li	LGPS/Li
MSD at 950 ps (Å^2)	2.01	13.45	57.96
Total reactive flux	0.29	0.44	0.45

Supplementary Table 4. Median residence diffusion coefficient of states (unit: Å^2/ps) in different SEI systems.

	LLZO/Li	LPSCl/Li	LGPS/Li
S0	5.82e-03	1.00e-02	9.78e-03
S1	1.60e-02	8.31e-03	1.04e-02
S2	3.00e-03	1.40e-02	1.15e-02
S3	1.78e-02	1.24e-02	1.09e-02
S4	3.02e-03	1.37e-02	1.53e-02

S5	1.29e-02	8.24e-03	9.46e-03

Supplementary Note 1

Silhouette score (s) was used to measure the cluster quality of lithium graphs. It was calculated through the scikit-learn package[1]:

$$s = \frac{(b-a)}{\max(a,b)} \tag{1}$$

Where a is the average intra-cluster distance, and b is the average nearest-cluster distance. Commonly, $s > 0.25$ indicates a reasonable classification.

Supplementary Note 2

For three SEI systems including LLZO/Li, LGPS/Li, and LPSCl/Li, we have total lithium fluxes f_{tot} and MSDs from each system denoted as $\{v_s\}$ and $\{u_s\}$. Pearson correlation $\gamma_{\text{flux}}(u,v)$ between these paired series $\{v_s\}$ and $\{u_s\}$ is calculated from:

$$\gamma_{\text{flux}}(u,v) = \frac{\sum_s (u_s-\bar{u})(v_s-\bar{v})}{\sqrt{\sum_s (u_s-\bar{u})^2}\sqrt{\sum_s (v_s-\bar{v})^2}} \tag{2}$$

where $\bar{u}$ and $\bar{v}$ are the sample means of $\{v_s\}$ and $\{u_s\}$ over the three systems. Spearman[2] rank correlation ρ_{flux} is obtained by applying this Pearson formula to the ranks of the two observables f_{tot} and MSD in each system:

$$\rho_{\text{flux}} = \gamma_{\text{flux}}\big(\text{rank}(f_{\text{tot}}), \text{rank}(\text{MSD})\big) \tag{3}$$

Supplementary Note 3

For the correlation between residence diffusion coefficient D_{res} and GET-SEI mobility μ_{m} in SEI systems, we calculate Spearman rank correlation ρ_{mob}:

$$\rho_{\text{mob}} = \gamma_{\text{mob}}\big(\text{rank}(D_{\text{res}}), \text{rank}(\mu_{\text{m}})\big) \tag{4}$$

Where γ_{mob} is the Pearson correlation (equation 2) between the paired series of D_{res} and μ_{m} denoted as $\{v_s\}$ and $\{u_s\}$.

Supplementary References

(1) Pedregosa, F.; Varoquaux, G.; Gramfort, A.; Michel, V.; Thirion, B.; Grisel, O.; Blondel, M.; Prettenhofer, P.; Weiss, R.; Dubourg, V. Scikit-learn: Machine learning in Python. *J. Mach. Learn. Res.* **2011**, *12*, 2825–2830.
(2) Spearman, C. The proof and measurement of association between two things. *Am. J. Psychol.* **1904**, *15*, 72–101.
